# Spatio-temporal expression profile of matrix metalloproteinase (Mmp) modulators Reck and Sparc during the rat ovarian dynamics

**DOI:** 10.1186/s12958-018-0422-2

**Published:** 2018-11-13

**Authors:** Gabriel Levin, Tatiane Maldonado Coelho, Nathali Guimarães Nóbrega, Marina Trombetta-Lima, Mari Cleide Sogayar, Ana Claudia Oliveira Carreira

**Affiliations:** 1 NUCEL (Cell and Molecular Therapy Center), Internal Medicine Department, Medical School, University of São Paulo, Rua Pangaré, 100, Cidade Universitária, São Paulo, SP 05360-130 Brazil; 2 Chemistry Institute, Biochemistry Department, University of São Paulo, São Paulo, SP 05508-000 Brazil; 3 Department of Surgery, School of Veterinary Medicine and Animal Science, University of São Paulo, São Paulo, SP 05508-270 Brazil

**Keywords:** Folliculogenesis, Ovary remodeling, Mmps system, Extracellular matrix, Reck, Sparc

## Abstract

**Background:**

Matrix metalloproteinases (Mmps) and their tissue inhibitors (Timps) are widely recognized as crucial factors for extracellular matrix remodeling in the ovary and are involved in follicular growth, ovulation, luteinization, and luteolysis during the estrous cycle. Recently, several genes have been associated to the modulation of Mmps activity, including *Basigin* (*Bsg)*, which induces the expression of Mmps in rat ovaries; *Sparc*, a TGF-β modulator that is related to increased expression of Mmps in cancer; and *Reck*, which is associated with Mmps inhibition. However, the expression pattern of Mmp modulators in ovary dynamics is still largely uncharacterized.

**Methods:**

To characterize the expression pattern of Mmps network members in ovary dynamics, we analyzed the spatio-temporal expression pattern of Reck and Sparc, as well as of Mmp2, Mmp9 and Mmp14 proteins, by immunohistochemistry (IHC), in pre-pubertal rat ovaries obtained from an artificial cycle induced by eCG/hCG, in the different phases of the hormone-induced estrous cycle. We also determined the gene expression profiles of *Mmps* (*2*, *9*, *13 14*), *Timps* (*1*, *2*, *3*), *Sparc*, *Bsg*, and *Reck* to complement this panel.

**Results:**

IHC analysis revealed that Mmp protein expression peaks at the early stages of folliculogenesis and ovulation, decreases during ovulation-luteogenesis transition and luteogenesis, increasing again during *corpus luteum* maintenance and luteolysis. The protein expression patterns of these metalloproteinases and Sparc were inverse relative to the pattern displayed by Reck. We observed that the gene expression peaks of Mmps inhibitors *Reck* and *Timp2* were closely paraleled by *Mmp2* and *Mmp9* suppression. The opposite was also true: increased *Mmp2* and *Mmp9* expression was concomitant to reduced *Reck* and *Timp2* levels.

**Conclusion:**

Therefore, our results generate a spatio-temporal expression profile panel of Mmps and their regulators, suggesting that Reck and Sparc seem to play a role during ovarian dynamics: Reck as a possible inhibitor and Sparc as an inducer of Mmps.

**Electronic supplementary material:**

The online version of this article (10.1186/s12958-018-0422-2) contains supplementary material, which is available to authorized users.

## Background

The ovarian dynamics comprehended by different phases of the estrous cycle involves extensive tissue remodeling [1], and major extracellular matrix (ECM) reorganization is a key part of this process. This tissue remodeling is dependent upon cyclic hormonal variation, proteins and peptide growth factors and is a pre-requisite for expansion of the growing ovulatory follicle, breakdown of follicular walls during ovulation, luteinization of the postovulatory follicles, and regression of the *corpus luteum* (CL) [2, 3]. Matrix metalloproteinases (Mmps), a large family of zinc-dependent proteases, and their also numerous inhibitors (Timps) play a major role in ovarian ECM reorganization [2–5]. Together, Mmps and Timps form a network that regulates tissue remodelling and is modulated not only by gonadotropins and steroid hormones, but, also, by the expression of other factors, such as TNF-α, IGF-1 [5–9], and Bsg, which is an inducer of Mmps during the luteinization process [10]. A complete map of the components of this network, as well as a precise characterization of their expression pattern, may allow us to better understand this tight regulation involved in ovarian tissue remodelling that occur during the estrous cycle.

It is already known from the literature that Mmp2, Mmp9, Mmp14 (Mt1-Mmp), and Mmp19 expression and activity in granulosa and/or theca cells are extensively linked to the disruption of ovarian ECM in rats, which is rich in collagen, laminin, and fibronectin, leading to follicular growth and release of the oocyte during the ovulatory process [11]. MMP1, MMP2 e MMP9 have been detected in cultures of luteinized granulosa cells [12–14].

The expression and activity of Mmps have been known to be upregulated by gonadotropins during these phases [15–19]. However, hCG also was shown to reduce the expression of MMP2 and MMP9 [12, 13]. Following ovulation, the ruptured follicle is transformed into a CL by extensive cellular reorganization, migration, and neovascularization [20–22], which is associated with notable gelatinolytic activity, whereas the regression and absorption of CL is marked by an increase in Mmp13 levels and activity [23, 24].

Tissue inhibitors of metalloproteinases (Timps) have also been extensively studied in ovaries during the estrous cycle. Timp1 is upregulated by gonadotropins during folliculogenesis and ovulation in rats [25–27], providing proteolytic homeostasis to the ECM, through inhibition of metalloproteinases in the corpus luteum, while Timp3 levels decrease slightly [28].

*Timp1* mRNA levels increase during luteal formation and regression [24, 29], while *Timp3* levels are elevated during luteal maintenance [29]. TIMP-2 has a role in the local regulation of MMP1 and MMP2 in the corpus luteum [30]. Moreover, in addition to these inhibitors, there are also proteins, which have already been described as being responsible for inducing the expression of Mmps and ensuring system homeostasis in the ovary. In this context, it is worth mentioning Basigin (Bsg), a protein associated with Mmps induction and also with tumor progression and endometriosis, being expressed in granulosa and theca cells of pre-ovulatory follicles, and also in CL [10, 31]. However, the expression pattern of Mmp modulators in ovary dynamics is still largely uncharacterized, and there is evidence that members of the network are still to be identified. In particular, two proteins implied in tumor aggressiveness and invasive potential, through modulation of ECM integrity, namely, Sparc and Reck, may have an important role in ovarian tissue remodeling, since they are expressed in ovarian cells and are involved in the regulation of the Mmp system in other tissues [32–36].

Sparc (secreted protein acid and rich in cysteine), also known as Osteonectin, a 43 K protein, and basement-membrane protein 40 (BM-40), is a calcium-binding matrix cellular protein that plays a role in matrix mineralization [37], modulates TGF-β [34], and is expressed in the internal theca and follicular basal lamina of ovine follicles and theca-derived small luteal cells [35]. Even though SPARC is highly expressed in several tumors, it may be able to inhibit tumorigenesis or tumor progression in human ovarian cancer [38] and its expression is reduced in ovarian cancer cell lines [38]. Additionally, Sparc is implicated in indirect Mmp modulation and turnover of many physiological processes, but it has not yet been described in whole ovary dynamics. Despite the fact that Bagavandoss et al. described Sparc in the ovary of the late stages of estrous cycle, its expression in the initial phases (folliculogenesis, ovulation and ovulation-luteinization transition) of this process is still unknown [32]. On the other hand, RECK (REversion-inducing-Cystein-rich protein with Kazal motifs) is a key regulator of extracellular matrix integrity and angiogenesis that is linked to the cell membrane by a GPI-anchor [36] and inhibits the activity of MMP2, MMP9, and MMP14 at different steps of their activation cascades in humans [36, 39]. *Reck* gene is expressed in several mouse tissues during development, including the uterus and ovaries, but at lower levels in most tissues, when compared to those of Timps 1, 2, 3, and 4 [33]. Reck expression is inhibited by estrogen within the mouse uterus, but this inhibition is partially blocked by progesterone [40]. To date, no information is available regarding the expression profile and function of Reck in rat ovaries development and maturation.

To analyse the ovarian extracellular matrix remodeling, we analyzed an artificial estral cycle, with a combination of equine chorionic gonadotropin (eCG) and human CG (hCG) in pre-pubertal rats [41], that have not yet started their estrous cycle, to induce multiple follicular development and ovulation. The rats only start their estrus cycles after the vaginal orifice opens, which tends to occur between postnatal 32 and 36 days [41]. The literature describes that the administration of 10UI eCG stimulates the folliculogenesis and after 48 h the injection of 10UI of hCG can induce ovulation [42, 43]. In sequence, it is observed formation of corpus luteum, and 24 and 48 h post-hCG injection, the luteal formation in the ovaries. After 4 and 8 days, a peak of progesterone production is maximal resulting in the luteal maintenance, and corpus luteum regression can be analyzed at 14 days.

Therefore, understanding Reck and Sparc expression during the estrous cycle is essential to understand which role, if any, they play in the regulation of ovarian tissue remodeling, which allows all of the complex physiological processes that take place in the ovary. In this context, our hypothesis is that the Mmps regulators Reck and Sparc are modulated in ovarian dynamics possibly controlling the expression of Mmps, Reck being a possible inhibitor and Sparc a possible inducer of Mmps in this process.

To address these questions, we characterized the spatio-temporal expression pattern of Reck and Sparc proteins, as well as of Mmp2, Mmp9 and Mmp14 by immunohistochemistry in pre-pubertal rat ovaries during different phases of the hCG/eCG induced estrous cycle. To complement these data, we used qRT-PCR to investigate *Mmps 2, 9, 13, 14, and 19, Timps 1, 2, and 3, Reck, Sparc, and Bsg* mRNA levels and generate a complete expression profile panel of these targets during induced folliculogenesis, ovulation, luteogenesis, and luteolysis in prepubertal rat ovaries.

## Methods

### Animal model

All animal experimentation was approved by the Ethics Committee for Animal Use of the Chemistry Institute (CEUA) on 08/28/2010, University of São Paulo, São Paulo, Brazil, in accordance with the National Council for the Control of Animal Experimentation (CONCEA).

To create an artificial estral cycle, we carried out the protocol to induce multiple follicular development and ovulation described by Jo and Curry and Ying and Meyer [42, 43]. First, 21 day-old pre-pubertal Sprague-Dawley female rats were induced by subcutaneous injection of 10 IU of equine chorionic gonadotropin (eCG) (Folligon, MSD Saúde Animal, São Paulo, SP, Brazil), resulting in animals in folliculogenesis phase. In sequence, ovulation and CL formation were induced by subcutaneous injection of 10 IU of human chorionic gonadotropin (hCG) (Choragon, Ferring GmbH, Kiel, Alemanha) 48 h after eCG injection. To analyze the relative expression pattern of the genes of interest throughout the hormonally induced estrous cycle, 10 experimental groups were established and different hormonal treatments were adopted to recreate the various stages of the estrous cycle. Treatment groups varied depending on the hormones applied (eCG only or both eCG and hCG) and induction and euthanasia periods of time. Animals were euthanized at 0, 24, or 48 h after eCG administration or 12, 24, or 48 h or 4, 8, or 14 days after hCG administration (Table 1). Two independent experiments were carried out, using three animals per experimental group. Animals injected with phosphate-buffered saline (PBS [137 mM NaCl, 2.7 mM KCl, 8 mM anhydrous Na_2_HPO_4_, and 1.4 mM KH_2_PO_4_; pH = 7.2]) were used as controls.

**Table 1:** Experimental groups and treatments

GROUP	10 IU eCG	10 IU hCG	PHASE
G1	0 h	–	Control
G2	24 h	–	Folliculogenesis
G3	48 h	–	
G4	48 h	12 h	Ovulation
G5	48 h	24 h	Transition: ovulation-luteinization
G6	48 h	48 h	Transition: ovulation-luteinization, *corpus luteum* formation
G7	48 h	4 days	*Corpus luteum* maintenance
G8	48 h	8 days	
G9	48 h	14 days	*Corpus luteum* regression

### Tissue samples

Both ovaries were collected from each animal. One ovary was stored in RNAholder solution (BioAgency Laboratories, São Paulo, SP, Brazil) at − 80 °C for total RNA extraction and the contralateral ovary was fixed in 4% paraformaldehyde solution (Sigma-Aldrich, St. Louis, MO, USA), stored at 4 °C, and subsequently included in paraffin for immunohistochemical analysis.

### Immunohistochemistry

**Fig. 1:**
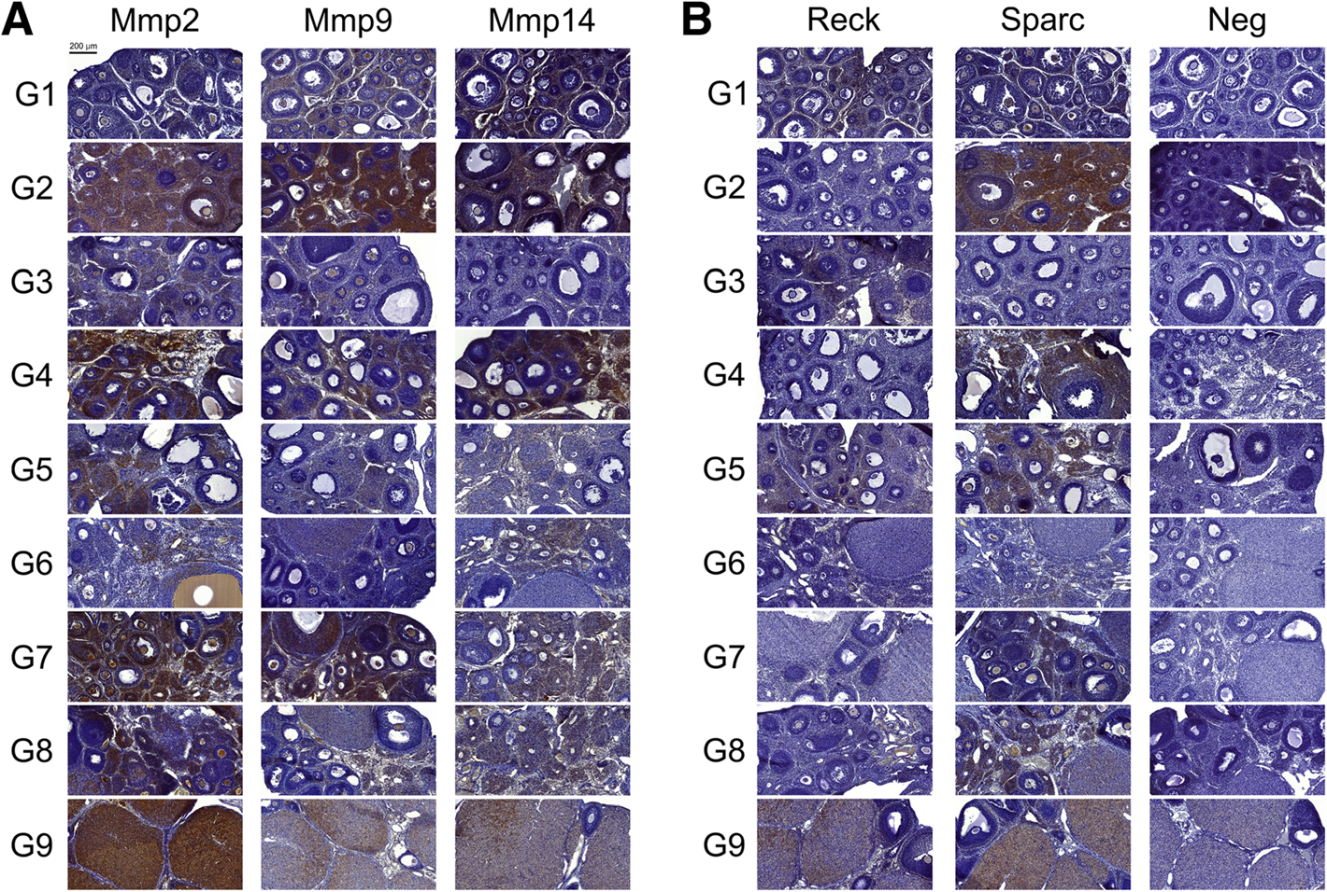
Immunohistochemistry (IHC) analysis of target proteins Mmp2, 9, 14, Reck, and Sparc during ovary dynamics across hormone-induced estrous cycle in rats. Brown staining indicates the presence of the protein. **a** Mmp2, Mmp9 and Mmp14; **b** Reck, Sparc and negative control (Neg)

The paraffin-embedded tissues were sectioned into 4 μm-thick sections using a microtome (Leica Biosystems, Wetzlar, HE, Germany) and placed onto polylysine-treated slides. The sections were then dewaxed in xylene (Synth, São Paulo, SP, Brazil), incubated with decreasing concentrations of ethanol (Synth), and rehydrated in PBS-T (0.1% Triton X-100 in PBS [123 mM NaCl, 9.7 mM Na_2_HPO_4_, and 51.1 mM KH_2_PO_4_; pH = 7.4]). Next, tissue antigens were retrieved using phosphate-citrate buffer (pH = 6.0; Sigma-Aldrich) at 98 °C for 20 min, slowly cooled down to room temperature, and washed in PBS-T. To block endogenous peroxidase, the tissues were incubated with 3% hydrogen peroxide at room temperature for 45 min, washed in PBS-T, and incubated for 20 min in 3% nonfat milk solution in PBS for blocking of non-specific sites. The tissues were individually incubated overnight with rabbit polyclonal antibodies against Mmp2 (1:1000 dilution, product no. ab79781; Abcam, Inc., Cambridge, MA, USA), Mmp9 (1:100 dilution, product no. ab38898; Abcam, Inc.), and Sparc (1:100 dilution, product no. #5420; Cell Signaling Technology, Danvers, MA, USA) or rabbit monoclonal antibodies anti-Mmp14 (1:100 dilution, product no. ab51074; Abcam, Inc.) and anti-Reck (1:200 dilution, product no. #3433; Cell Signaling Technology) at 4 °C. Information on the antibodies and dilutions used in the IHC experiments are summarized in Additional file 1: Table S1. Antibody dilutions were made in PBS with 0.1% bovine serum albumin (BSA). The signal was amplified using the labeled streptavidin-biotin (LSAB) method with the Universal Dako LSAB + peroxidase kit (Dako North America, Inc., Via Real Carpinteria, CA, USA). Tissues were incubated for 30 min with the biotinylated link, washed twice with PBS-T, and incubated with streptavidin conjugated to horseradish peroxidase. For the IHC-negative control, the primary antibody was omitted. Tissues were revealed using the DAB kit (Dako North America) and counterstained with Harris hematoxylin (Easypath, São Paulo, SP, Brazil). Lastly, the sections were dehydrated, xylene embedded, and assembled with VectaMount permanent mounting medium (Vector Laboratories, Burlingame, CA, USA). Immunohistochemically stained sections were scanned using the Pannoramic P250 Flash II Slide Scanner (3DHISTECH, Budapest, Hungary), and the images were selected using the CaseViewer software (3DHISTECH, version 2.0). Representative areas of the tissue were selected for each condition to compose Fig. 1 and IHC scores were generated after analysis by three independent researchers (Table 2). IHC kits were provided by Dako North America.

**Table 2:** Intensity and localization of target proteins in ovarian tissue during hormone-induced estrous cycle

GROUP	PHASE	Mmp2	Mmp9	Mmp14	Reck	Sparc
PBS	Vehicle control	(4) G^a^/T/E/O	(2) E	(2) E	(2) T/E	(2) T/E/O
			(3) O			
G1	Control	(2) T/O	(3) T/E/O	(3) T/E	(3) T/E	(2) T/E/O
G2	Folliculogenesis	(4) G^a^/T/E/O	(5) G^a^/T/E/O	(4) T/E	0	(4) G^a^/T/E/O
G3		(3) T/E/O	(2) E	0	(2) T/E	0
			(3) O			
G4	Ovulation	(5) T/E/O	(4) G^a^/T/E/O	(5) G^a^/T/E	0	(4) T/E/O
G5	Transition: ovulation-luteinization	(4) T/E/O	(2) T/E/O	(2) E	(3) T/E	(3) T/E/O
G6	Transition: ovulation-luteinization, luteinization	(3) CL/T/E/O	(2) CL/E/O	(2) E	(1) CL	(2) CL/E/O
G7	CL maintenance	(5) CL/G^a^/T/E/O	(5) CL	(3) T/E	(1) CL/T/E	(3) CL/T/E/O
			(4) G^a^/T/E/O			
G8		(4) CL/T/E/O	(2) CL	(2) E	(2) T/E	(3) CL/T/E/O
			(3) G^a^/T/E/O			
G9	Luteolysis	(6)	(3) CL/O	(4) CL	(3) CL/T/E	(4) CL
		CL/G^a^/T/E/O		(3) T/E		(2) T/E/O

Localization is identified as: CL: *corpus luteum*; T: theca; G: granulosa; E: stroma; O: oocyte. Staining intensity (0–6): unstained (0), low staining (1–2), moderate staining (3–4), and strong staining (5–6)

^a^Expression unevenly detected across the tissue

### RNA isolation, cDNA synthesis, and reverse transcriptase PCR

Total RNA was isolated using the TRIzol (Invitrogen, Carlsbad, CA, USA) protocol. RNA quality was evaluated by the spectrophotometric absorbance ratios at 280/260 nm and 230/260 nm (Nanodrop, Thermo Scientific, Waltham, MA, USA). Equal amounts of RNA treated with DNaseI (Thermo Scientific) from each of the three ovaries were mixed into a single pool for reverse transcription. Complementary DNA was synthetized from 2μg of total RNA pool using ImProm-II Reverse Transcriptase (Promega, Madison, WI, USA) according to the manufacturer’s instructions.

### qRT-PCR

The relative mRNA expressions of the target genes were assessed using the Maxima SYBR Green/ROX qPCR Master Mix (Thermo Scientific) in a GeneAmp 7300 Sequence Detection System (Applied Biosystems, Foster City, CA, USA), according to the manufacturer’s instructions. The primers used to amplify the *Mmps* (*2*, *9*, *13*, *14*, and *19*), *Timps* (*1*, *2*, and *3*), *Sparc*, *Bsg*, and *Reck* genes are listed in Additional file 1: Table S2. A dissociation cycle was performed after each run to check for non-specific amplification. Relative mRNA expression levels were estimated using the method described in [44], which generates a normalization value in geNorm Software using *β-Actin* and *Hprt* as housekeeping genes.

### Statistical analysis

Relative mRNA expression levels across treatment groups were normalized relative to the average expression of control animals treated with saline solution and compared using One-way analysis of variance (ANOVA), followed by Dunnett’s post hoc comparison test. Spearman correlations were calculated for each gene pair (Additional file 1: Table S3). All analyses were carried out using the SPSS Software (IBM SPSS Statistics; version 20). A *p* value of *p* < 0.05 was considered significant (* *p* < 0.05, ** *p* < 0.01, and *** *p* < 0.001).

## Results

### Expression profile of Mmps during ovary ECM remodeling across hormone-induced estrous cycle

We studied the spatio-temporal patterns of expression of Mmps 2, 9, and 14 proteins within the ovarian tissue using immunohistochemistry. Figure 1a shows representative images for each condition, with the target staining intensities and respective localizations being summarized in Table 2. All Mmps analyzed were mainly restricted to the theca and stromal cells, except during the late stages of the estrous cycle (G7 and G8), when expression was also detected in the *corpus luteum*. Oocytes and granulosa cells were immune-reactive to gelatinases only. Ovaries showed a global increase in Mmp protein expression during the early stages of folliculogenesis (G2), followed by another expression peak during ovulation (G4). Nevertheless, the expression levels of gelatinases were lower during the ovulation-luteogenesis transition and CL formation and increased during CL maintenance, more specifically, at luteolysis.

**Fig. 2:**
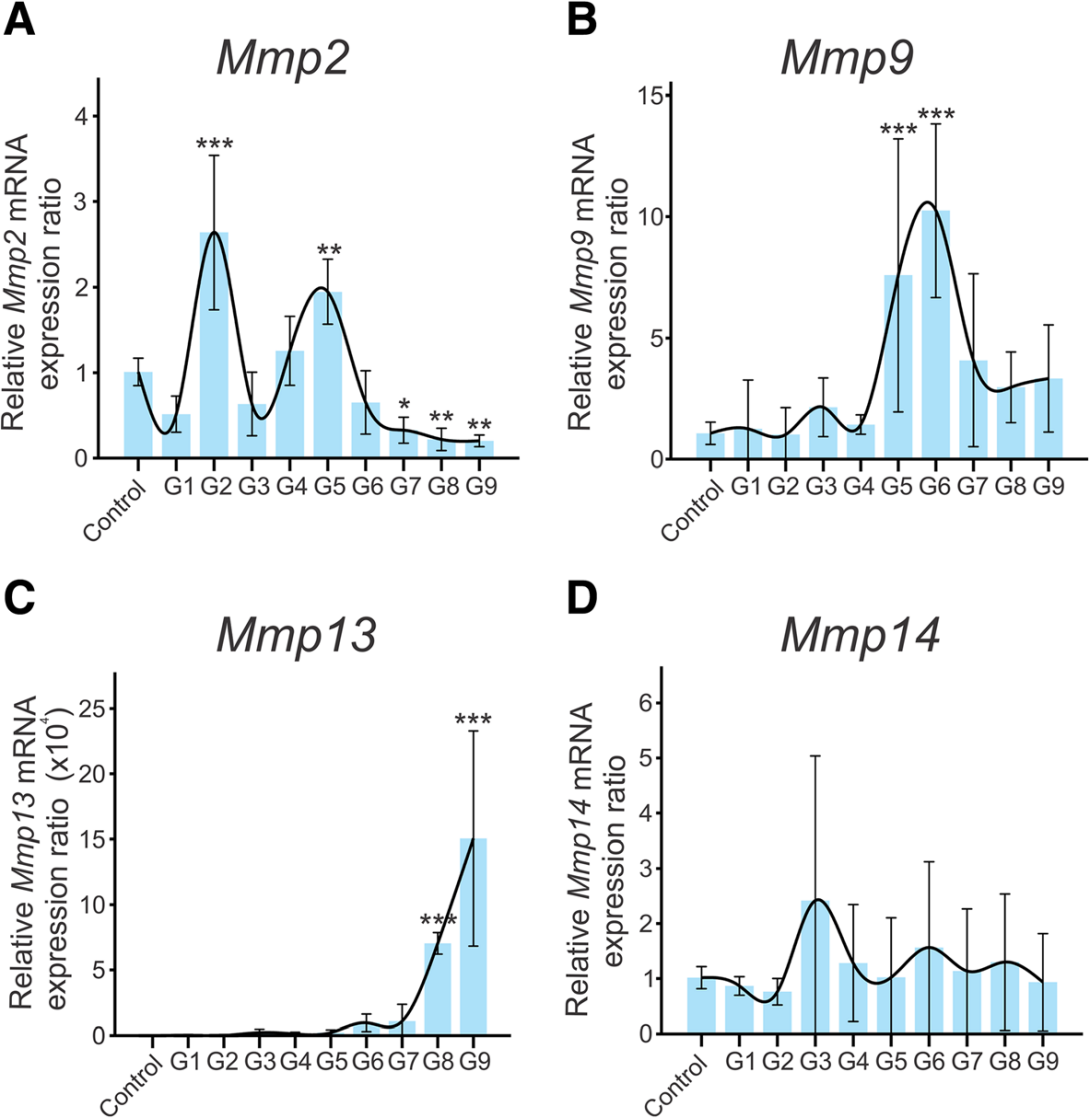
mRNA expression profile of matrix metalloproteinases (*Mmps*) during ovary dynamics across hormone-induced estrous cycle in rats. (**a**) *Mmp2*, (**b**) *Mmp9*, (**c**) *Mmp13* and (**d**) *Mmp14*. Expression levels were normalized to the average expression of control animals treated with saline solution. The statistical significances were relative to control group. * *Mmp2* expression level was lower in the *corpus luteum* (G7) (*p* < 0.05). ** *Mmp2* levels were significantly higher at the ovulation-luteogenesis transition phase (G5) and lower at late stages of the CL maintenance and luteolysis G8-G9 (*p* < 0.01). *** *Mmp2* levels were significantly higher in early folliculogenesis ovaries (G2) than in ovaries from the control group; *Mmp9* levels were higher at ovulation-luteinization Transition (G5) and luteinization (G6); *Mmp13* were levels higher at late stages of the CL maintenance and luteolysis (G8-G9) (*p* < 0.001)

Figure 2 shows the relative expression pattern of *Mmps,* at the mRNA level, by qRT-PCR. *Mmp2* mRNA levels were significantly lower in ovaries from the control group, when compared to the early folliculogenesis ovaries (G2), displaying a peak at the ovulation-luteogenesis transition phase (G5) followed by a sharp decrease (Fig. 2a). Similarly, *Mmp9* rapidly increased, peaked at G5 and decreased after G6 (Fig. 2b). Meanwhile, *Mmp13* peaked at late stages of the G8-G9 protocol (Fig. 2c). *Mmp14* (Fig. 2d) and *Mmp19* were not modulated during the process (data not shown).

### Expression profile of the Mmps’ modulators Reck, Bsg and Sparc during ovary ECM remodeling across hormone-induced estrous cycle

**Fig. 3:**
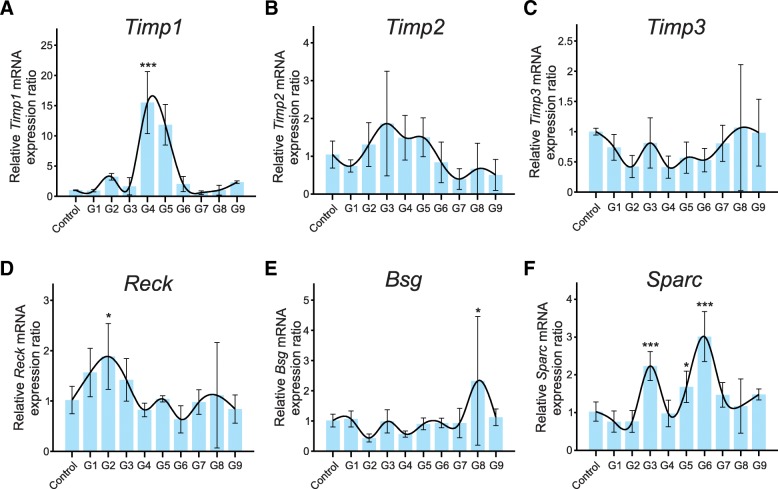
Mmps modulators mRNA expression profile of matrix metalloproteinase (*Mmp*) modulators during ovary dynamics across hormone-induced estrous cycle in rats. Mmp inhibitors: (**a**) *Timp1*, (**b**) *Timp2*, (**c**) *Timp3*, (**d**) *Reck*; Mmp inducers: (**e**) *Bsg* and (**f**) *Sparc*. Expression levels were normalized to the average expression of control animals treated with saline solution. The statistical significances were relative to control group. * Reck level was significantly higher at folliculogenesis phase (G2); *Bsg* level was significantly higher during the late stage of *corpus luteum* maintenance (G8); *Sparc* levels were significantly higher at late folliculogenesis (G3) and at ovulation-luteogenesis transition to *corpus luteum* maintenance (G6) (*p* < 0.05). *** *Timp1* level was significantly higher during ovulation (G4 and G5); *Sparc* levels were higher at late folliculogenesis (G3) and at ovulation-luteogenesis transition to *corpus luteum* maintenance (G5 and G6) (*p* < 0.001)

Figure 3 presents the mRNA expression profile of different Mmps modulators during the hormone-induced estrous cycle, the inhibitors *Timps* (*Timp1*, *Timp2*, and *Timp3*) (Fig. 3a, B, and C, respectively) and *Reck* (Fig. 3d) and the *Mmps* inducers *Bsg* and *Sparc* (Fig. 3e and f, respectively) in whole ovarian RNA extracts*. Timp1* mRNA showed a clear expression peak during ovulation (G4 and G5, Fig. 3a), while *Timp2* and *Timp3* apparently are not significantly modulated in this model (Fig. 3b and c). Expression levels were normalized relative to the average expression of control animals treated with saline solution.

As may be observed in Fig. 1b and Table 2, the Reck protein, the only Mmp inhibitor which is anchored to the membrane by a glycosyl phosphatidyl anchor, was expressed in theca cells, ovarian stroma, and CL, but the protein expression pattern was opposite to that of Mmps, namely: no Reck protein expression was detected during early folliculogenesis and ovulation, while the highest Reck levels occurred immediately following eCG administration (G1) and during ovulation-luteogenesis transition (G5). The only exception was luteolysis (G9), during which Reck protein and the Mmps were both detected at high levels. Additionally, *Reck* mRNA was upregulated at the early stages of the estrous cycle during folliculogenesis, with an expression peak at G2 (Fig. 3d). Furthermore, its gene expression negatively correlated with *Mmp13* collagenase expression (Additional file 1: Table S2). Conversely, the Mmp inducer *Bsg* displayed an mRNA expression peak during the late stage of CL maintenance (G8) (Fig. 3e).

The ovarian tissue expressed the Sparc protein in theca, granulosa and stromal cells, in oocytes and CL. The Sparc modulation pattern across the estrous cycle was similar to that of Mmps, with protein expression peaks during early folliculogenesis (G2), ovulation (G4), and luteolysis (G9), and lower expression levels between these phases (Fig. 1b, Table 2). High *Sparc* mRNA expression was observed at late folliculogenesis (G3) and from ovulation-luteogenesis transition to CL maintenance (G5 and G6, Fig. 3f).

## Discussion

In this study, we evaluated the spatio-temporal expression pattern of several Mmps and their regulating proteins in whole ovaries at several stages of the estrous cycle using immunohistochemistry to generate a panel of these players in rat ovarian dynamics. In addition, we also quantitatively assessed the mRNA expression of *Mmps* and their regulators at those same estrous cycle stages. Interestingly, some of the analyzed genes (*Mmp9, Reck* and *Sparc*) displayed a discrepancy between the observed mRNA and protein levels at specific phases of the induced estrous cycle. A poor correlation between mRNA and protein levels has been extensively reported highlighting the importance of an integrative approach [45–47] or the development of a gene specific RNA-to-protein conversion factor [48]. Particularly, MMPs and their inhibitors have been shown to display discrepancies in their mRNA to protein correspondence in human prostate tissue and in skeletal muscle cancer in cachexia-associated matrix remodeling [49, 50] and further studies are necessary to identify the mechanisms that lead to this disparity for this particular targets.

Analysis of Mmp spatial and temporal protein distribution, through immunohistochemistry, revealed that the Mmp2 and Mmp9 gelatinases display an overall higher expression during the rat estrous cycle, when compared to the Mmp14 collagenase. Gelatinases had a widespread spatial distribution in the ovaries, being detected in the theca, stromal, and granulosa cells, oocytes and CL. Mmp14 protein expression, on the other hand, was mainly restricted to theca and stromal cells, but at late stages of the estrous cycle, during luteolysis (G9), being also detected at the CL. The *Mmp14* transcript levels apparently did not vary across ovulation and CL lifespan, similarly to what has been described in the literature [24, 51]. Nevertheless, high Mmp14 protein levels were detected in CL during its regression, indicating a possible contribution of this MMP to local tissue remodeling.

*Mmp2* mRNA levels displayed a 2.8-fold increase during early folliculogenesis (G2), followed by a reduction (G3) and displayed a peak during the ovulation-luteogenesis transition phase (G5). At this same stage (G5), 24 h after hCG injection, *Mmp9* expression displayed an increase, peaking at luteinization (G6). Furthermore, both gelatinases displayed a reduced mRNA expression during CL maintenance (G7-G8) and regression (G9). Our results support previously published gene expression data from other groups, corroborating the increased gelatinolytic and collagenolytic activity during folliculogenesis and *Mmp2* and *Mmp9* expression 24 h post-hCG injection (luteogenesis, G5) in other studies [16, 23, 24, 51], indicating a role for this enzyme in the tissue remodeling associated with luteolysis.

We observed an increase in *Mmp13* mRNA expression during the hormone-induced estrous cycle, which reached a peak of 150,000-fold 14 days after hCG injection (G9), suggesting that this gene is strongly correlated with luteal regression. These data are supported by others: Nothnick et al. [23] observed a 15-fold increase in *Mmp13* mRNA expression 12 days after hCG and Liu et al. [24], using an adult pseudo-pregnant rat model, also showed an increase in *Mmp13* expression during luteal regression [24].

Our data suggest that Mmp expression is tightly related to the main biological events taking place during the estrous cycle, supporting previously published data and adding evidence to the importance of proteolytic tissue-degradation for ovulation in primates and rodents [52]. Equally important as determining Mmps expression during the estrous cycle is to evaluate the expression of their inhibitors and inducers, since the balance between all of these proteins is what determines the extent and nature of ECM remodeling.

*Timp1* was the only member of the Timp family to show significant mRNA modulation in this work, presenting a sharp increase in mRNA levels during ovulation (G4), 12 h after hGC administration. Conversely, for an Mmp inhibitor, its expression positively correlates with *Mmp2* gene expression. Li and Curry [53] reported that the expression of both *Timp1* and *Timp3* mRNAs was induced upon treatment with hCG in pre-ovulatory rat granulosa cells in an epidermal growth factor receptor tyrosine kinase and mitogen-activated protein kinase (MAPK) pathway-dependent fashion [53]. This differential regulation is of extreme interest because Timps are multifunction proteins, whose Mmp-inhibition independent functions might have an important role in this model.

Takahashi and colleagues observed that the RECK MMP inhibitor has a privileged peri-cellular localization in human cells, due to its anchoring to the cell membrane by GPI [36]. Although *RECK* gene is widely expressed in normal organs [36], no description is available to date about its expression pattern in ovaries, despite the intense MMP-directed ECM remodeling that occurs in these organs. Our analysis of Reck expression in rat ovarian dynamics, by immunohistochemistry, showed no detectable Reck protein expression in early folliculogenesis (G2) and ovulation (G4), while Mmps 2, 9 and 14 levels were elevated. Following the same pattern, Reck protein is expressed at detectable levels in theca and stromal cells at the end of folliculogenesis (G3) whereas expression of Mmps is lower. In addition, during the ovulation-luteogenesis transition phase (G5), an increase in Reck protein expression in theca and stromal cells was observed in parallel to a decrease in Mmp9 expression, but not of Mmp2, in the same cells. Also, at late stages of ovarian remodeling, Reck was expressed in the *corpus luteum*, during luteogenesis, CL maintenance and regression (G6-G9). Moreover, *Reck* mRNA reached a significant expression peak during the late stages of folliculogenesis (G3), 48 h post-eCG stimulus, opposite to the reduction observed in gelatinases mRNA levels at this point. This orchestrated modulation of Reck and Mmps expression suggests that Reck may be involved in Mmps modulation in this model. In support of this hypothesis, the literature describes that RECK controls MMPs 2, 9, and 14 activities through several mechanisms, including inhibition of their proteolytic activity and activation cascade, as well as their sequestration [36, 39, 54].

Regarding Mmp inducers, our gene expression analysis shows that *Bsg* mRNA levels peak at CL manteinance (G8). In agreement, other authors used a rat hormone-induced estrous cycle model to determine a *Bsg* mRNA expression panel, observing that this gene displayed increased expression during luteinization, which persisted until CL regression [10]. Sparc, another Mmp inducer, was also investigated, revealing a spatio-temporal pattern modulation in rat ovaries during the estrous cycle. Spatial and temporal expression of Sparc protein was widespread, similarly to the pattern observed for Mmps. The Sparc protein profile was the similar to that of Mmps and opposite to the one displayed by Reck. According to Bagavandoss and collaborators, Sparc protein has been detected in theca and interstitial cells and its expression has been shown to increase in rat granulosa cells following hCG administration, during the final phases of ovarian development [32]. However, in the current study, we also detected Sparc protein expression in theca, stromal, granulosa cells and oocytes in the whole hormone-induced estrous cycle, except at late folliculogenesis. It is important to note that this is the first time that Sparc protein was observed in oocytes and in the initial stages of the ovarian remodeling, namely folliculogenesis, ovulation and ovulation-luteinization transition in rat model. In addition, gene expression analyses indicate high levels of relative *Sparc* mRNA expression during late folliculogenesis (G3) and ovulation-luteogenesis transition (G5, G6). Taken together, these results suggest that the Sparc transcript and protein may also play a role in the estrous cycle. In this same direction, Smith et al., using an ovine model, suggested that *SPARC* gene is an important modulator of ovarian development and dynamics which is involved in tissue reconstruction during pregnancy [35].

Therefore, our results generate an expression profile panel of Mmps and their regulators, suggesting that Reck and Sparc seem to play an important role at specific steps of the process, being part of a larger picture in rat ovarian dynamics.

## Conclusions

**Fig. 4:**
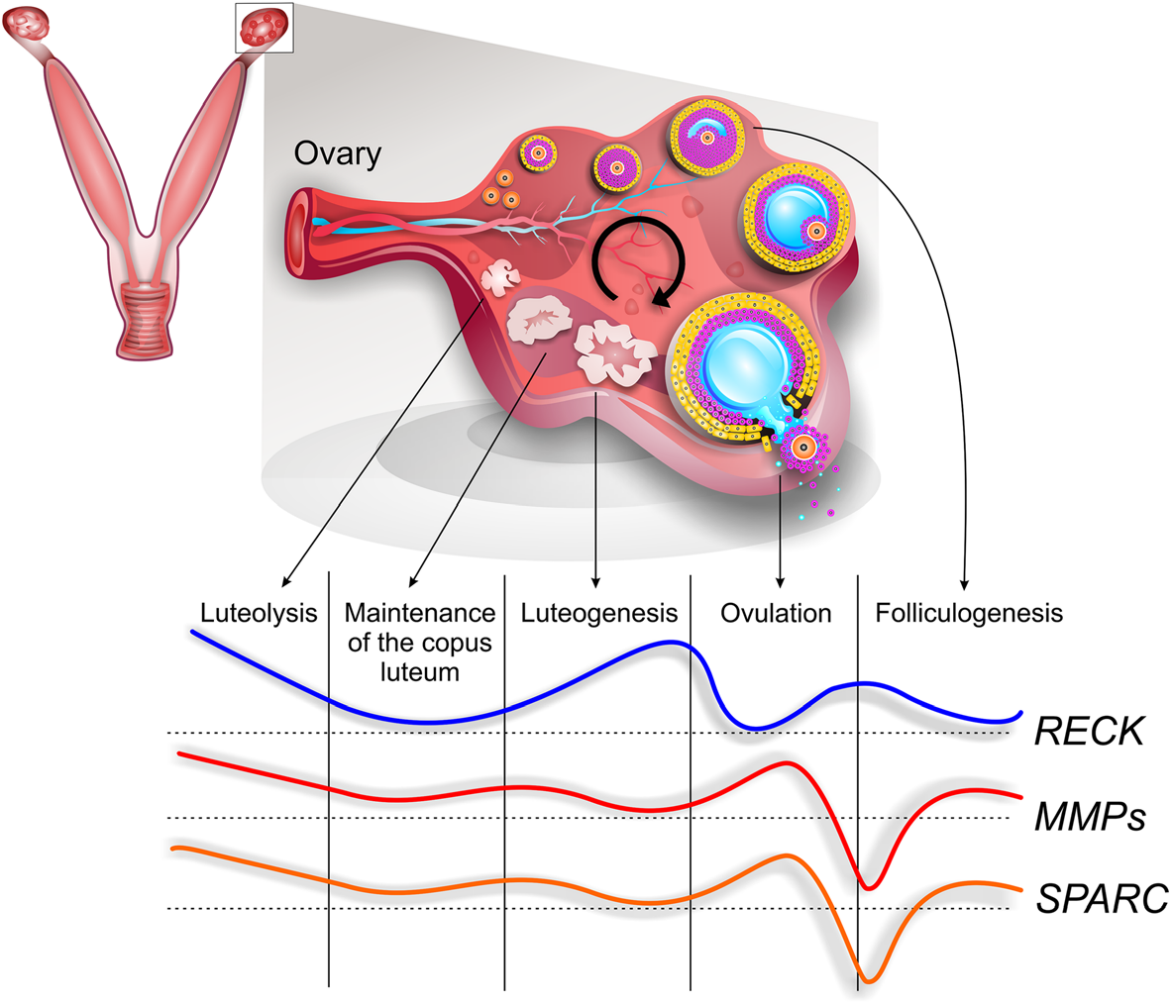
Global schematic view of matrix metalloproteinases (Mmps) and protein expression profile of their modulators during hormone-induced estrous cycle in prepubescent rats. Note the apparent inverse expression pattern of Mmps inhibitor Reck compared to Mmps and its inducer Sparc

Here we present a global view of Mmps and their regulators during a hormone-induced estrous cycle in pre-pubertal rats (Fig. 4). Our results show that both Reck and Sparc are differentially expressed in the rat ovary model, suggesting that these proteins may play important roles in ovary ECM remodeling. For the first time, a spatio-temporal profile of Reck and Sparc was described, evidencing an opposite regulation pattern between these Mmps regulators, with Reck as a possible inhibitor and Sparc as an inducer of Mmps, during ovarian dynamics.

## Additional file

Additional file 1: **Table S1.** Primary antibody characteristics and concentrations used in IHC assays. **Table S2.** Sequence and concentration of primers used in qRT-PCR assays. **Table S3.** Spearman correlation coefficient between the expression profiles of all analyzed genes. (DOC 90 kb)

## Abbreviations

Bsg: Basigin
CL: Corpus Luteum
eCG: Equine chorionic gonadotropin
ECM: Extracellular matrix
hCG: Human chorionic gonadotropin
IHC: Immunohistochemistry
IU: International unit
Mmps: Matrix metalloproteinases
PBS: Phosphate-buffered saline
PBS-T: 0.1% Triton X-100 in Phosphate-buffered saline
Reck: Reversion-inducing-Cystein-rich protein with Kazal motifs
Sparc: Secreted protein acid and rich in cysteine
Timps: Tissue inhibitors of metalloproteinases
