# Supplementary material for: Spatio-temporal expression profile of matrix metalloproteinase (Mmp) modulators Reck and Sparc during the rat ovarian dynamics
Source: Reprod Biol Endocrinol. 2018 Nov 13;16:116. doi: 10.1186/s12958-018-0422-2 (PMC6234678; doi:10.1186/s12958-018-0422-2)
Supplement: Supplementary file 1 — Table S1. Primary antibody characteristics and concentrations used in IHC assays. Table S2. Sequence and concentration of primers used in qRT-PCR assays. Table S3. Spearman correlation coefficient between the expression profiles of all analyzed genes. (DOC 90 kb) [file 12958_2018_422_MOESM1_ESM.doc]

**Table S1.** Primary antibody characteristics and concentrations used in IHC assays.

| Antibody | Description | Concentration | Clone | Catalogue Number | Source |
| --- | --- | --- | --- | --- | --- |
| Anti-Mmp2 | Rabbit polyclonal | 1:1000 | NA | ab79781 | Abcam, Inc. |
| Anti-Mmp9 | Rabbit polyclonal | 1:100 | NA | ab38898 | Abcam, Inc. |
| Anti-Mmp14 | Rabbit monoclonal | 1:100 | EP1264Y | ab51074 | Abcam, Inc. |
| Anti-Reck | Rabbit monoclonal | 1:200 | D8C7 | #3433 | Cell Signaling Technology |
| Anti-Sparc | Rabbit polyclonal | 1:100 | NA | #5420 | Cell Signaling Technology |

**Table S2.** Sequence and concentration of primers used in qRT-PCR assays.

| ***Primer*** | **Concentration** | **Forward Sequence** | **Reverse Sequence** |
| --- | --- | --- | --- |
|  | **(nM)** |  |  |
| *Mmp2* | 200 | CGATGTCTCCCCCAAAACAG | GCAGCCATAGAAAGTGTTCAGGTA |
| *Mmp9* | 400 | CGACATAAAAGGCATCCAGCAT | GGAGGCCTTGGGTCAGGTT |
| *Mmp13* | 200 | CCCCTTCCCTATGGTGATGA | CAAGTAGTGCTCTGCAAACTCAAGA |
| *Mmp14* | 200 | CAAAGGTTCTATGGTTTACAAGTGACA | CGCCTCATAGCCTTCATCGT |
| *Mmp19* | 200 | GGAGCTGATGACTTCAGGCTAGA | CAGTTCAGATGCTTCCTGGAAA |
| *Timp1* | 100 | ACCCACCCACAGACAGCTTT | GGAACCCATGAATTTAGCCCTTA |
| *Timp2* | 200 | CAGGCGTTTTGCAATGCA | CCTCCTTCTCGCTCACTGCTT |
| *Timp3* | 200 | CCAGGATGCCTTCTGCAACT | GCTTCTTTCCCACCACTTTGG |
| *Bsg* | 200 | GCGGCGGGCACCAT | AAGCAGGTAAGCTGTGTCTTGGA |
| *Reck* | 200 | AAAGTTTGCAGGAAAGAATATGAGAAC | CACACCGAGCCCATTTCATT |
| *Sparc* | 400 | CCTGGATCTTCTTTCTCCTTTGC | TTTCCTCCGCCACCATCTC |
| *β-Actin** | 100 | ACCCGCGAGTACAACCTTCTT | TATCGTCATCCATGGCGAACT |
| *Hprt** | 200 | CCGACCGGTTCTGTCATGTC | CATAACCTGGTTCATCATCACTAATCA |

*Reference Genes

|  | | *Mmp2* | *Mmp9* | *Mmp13* | *Mmp14* | *Mmp19* | *Timp1* | *Timp2* | *Timp3* | *Reck* | *Sparc* | | *Bsg* |  |
| --- | --- | --- | --- | --- | --- | --- | --- | --- | --- | --- | --- | --- | --- | --- |
| *Mmp2* | C | 1 | -0.617 | -0.5 | 0.417 | 0.3 | .767^*^ | .917^**^ | 0.267 | .717^*^ | 0.017 | | 0.217 |  |
|  | S | . | 0.077 | 0.17 | 0.265 | 0.433 | 0.016 | 0.001 | 0.488 | 0.03 | 0.966 | | 0.576 |  |
| *Mmp9* | C | -0.617 | 1 | .800^**^ | -0.633 | -0.333 | -0.383 | -.717^*^ | -0.167 | -0.567 | 0.433 | | -0.033 |  |
|  | S | 0.077 | . | 0.01 | 0.067 | 0.381 | 0.308 | 0.03 | 0.668 | 0.112 | 0.244 | | 0.932 |  |
| *Mmp13* | C | -0.5 | .800^**^ | 1 | -0.483 | -0.133 | -0.117 | -0.567 | -0.433 | -.767^*^ | 0 | | -0.067 |  |
|  | S | 0.17 | 0.01 | . | 0.187 | 0.732 | 0.765 | 0.112 | 0.244 | 0.016 | 1 | | 0.865 |  |
| *Mmp14* | C | 0.417 | -0.633 | -0.483 | 1 | 0.583 | 0.133 | 0.317 | 0.317 | 0.433 | -0.45 | | 0.017 |  |
|  | S | 0.265 | 0.067 | 0.187 | . | 0.099 | 0.732 | 0.406 | 0.406 | 0.244 | 0.224 | | 0.966 |  |
| *Mmp19* | C | 0.3 | -0.333 | -0.133 | 0.583 | 1 | 0.317 | 0.233 | 0.433 | 0.067 | -.667^*^ | | -0.3 |  |
|  | S | 0.433 | 0.381 | 0.732 | 0.099 | . | 0.406 | 0.546 | 0.244 | 0.865 | 0.05 | | 0.433 |  |
| *Timp1* | C | .767^*^ | -0.383 | -0.117 | 0.133 | 0.317 | 1 | .817^**^ | 0.033 | 0.25 | -0.05 | | 0.317 |  |
|  | S | 0.016 | 0.308 | 0.765 | 0.732 | 0.406 | . | 0.007 | 0.932 | 0.516 | 0.898 | | 0.406 |  |
| *Timp2* | C | .917^**^ | -.717^*^ | -0.567 | 0.317 | 0.233 | .817^**^ | 1 | 0.05 | 0.55 | -0.033 | | 0.2 |  |
|  | S | 0.001 | 0.03 | 0.112 | 0.406 | 0.546 | 0.007 | . | 0.898 | 0.125 | 0.932 | | 0.606 |  |
| *Timp3* | C | 0.267 | -0.167 | -0.433 | 0.317 | 0.433 | 0.033 | 0.05 | 1 | .667^*^ | 0.117 | | 0.233 |  |
|  | S | 0.488 | 0.668 | 0.244 | 0.406 | 0.244 | 0.932 | 0.898 | . | 0.05 | 0.765 | | 0.546 |  |
| *Reck* | C | .717^*^ | -0.567 | -.767^*^ | 0.433 | 0.067 | 0.25 | 0.55 | .667^*^ | 1 | 0.267 | | 0.333 |  |
|  | S | 0.03 | 0.112 | 0.016 | 0.244 | 0.865 | 0.516 | 0.125 | 0.05 | . | 0.488 | | 0.381 |  |
| *Sparc* | C | 0.017 | 0.433 | 0 | -0.45 | -.667^*^ | -0.05 | -0.033 | 0.117 | 0.267 | 1 | | 0.583 |  |
|  | S | 0.966 | 0.244 | 1 | 0.224 | 0.05 | 0.898 | 0.932 | 0.765 | 0.488 | . | | 0.099 |  |
| *Bsg* | C | 0.217 | -0.033 | -0.067 | 0.017 | -0.3 | 0.317 | 0.2 | 0.233 | 0.333 | 0.583 | | 1 |  |
|  | S | 0.576 | 0.932 | 0.865 | 0.966 | 0.433 | 0.406 | 0.606 | 0.546 | 0.381 | 0.099 | | . |  |
| *. Correlation is significant at the 0.05 level (2-tailed). | | | | | | | | | | | |  | | |
| **. Correlation is significant at the 0.01 level (2-tailed).  C= Correlation Coefficient  S= Sig. (2-tailed) | | | | | | | | | | | |  | | |

**Table S3.** Spearman correlation coefficient between the expression profiles of all analyzed genes.
